# On the Variability of Heart Rate Variability—Evidence from Prospective Study of Healthy Young College Students

**DOI:** 10.3390/e22111302

**Published:** 2020-11-15

**Authors:** Xingran Cui, Leirong Tian, Zhengwen Li, Zikai Ren, Keyang Zha, Xinruo Wei, Chung-Kang Peng

**Affiliations:** 1 Key Laboratory of Child Development and Learning Science, Ministry of Education, School of Biological Science & Medical Engineering, Southeast University, Nanjing 210096, China; 220195132@seu.edu.cn (L.T.); 213172473@seu.edu.cn (Z.L.); 213172649@seu.edu.cn (Z.R.); 213170049@seu.edu.cn (K.Z.); 213172511@seu.edu.cn (X.W.); 2 Institute of Biomedical Devices (Suzhou), Southeast University, Suzhou 215000, China; 3 Center for Dynamical Biomarkers, Division of Interdisciplinary Medicine and Biotechnology, Beth Israel Deaconess Medical Center, Harvard Medical School, 330 Brookline Avenue, Boston, MA 02215, USA; ckpeng@comcast.net

**Keywords:** heart rate variability, entropy, healthy young college students, ECG recording conditions, standardization

## Abstract

Heart rate variability (HRV) has been widely used as indices for autonomic regulation, including linear analyses, entropy and multi-scale entropy based nonlinear analyses, and however, it is strongly influenced by the conditions under which the signal is being recorded. To investigate the variability of healthy HRV under different settings, we recorded electrocardiograph (ECG) signals from 56 healthy young college students (20 h for each participant) at campus using wearable single-lead ECG device. Accurate R peak to R peak (RR) intervals were extracted by combing the advantages of five commonly used R-peak detection algorithms to eliminate data quality influence. Thorough and detailed linear and nonlinear HRV analyses were performed. Variability of HRV metrics were evaluated from five categories: (1) different states of daily activities; (2) different recording time period in the same day during free-running daily activities; (3) body postures of sitting and lying; (4) lying on the left, right and back; and (5) gender influence. For most of the analyzed HRV metrics, significant differences (*p* < 0.05) were found among different recording conditions within the five categories except lying on different positions. Results suggested that the standardization of ECG data collection and HRV analysis should be implemented in HRV related studies, especially for entropy and multi-scale entropy based analyses. Furthermore, this preliminary study provides reference values of HRV indices under various recording conditions of healthy young subjects that could be useful information for different applications (e.g., health monitoring and management).

## 1. Introduction

Heart rate variability (HRV) refers to the beat-to-beat variation of the time interval from R peak to R peak (i.e., RR intervals, RRIs or inter-beat intervals, IBIs) in the electrocardiograph (ECG) signals. Healthy heart rate fluctuates in complex patterns, driven by the complex nonlinear interactions of two competing forces (sympathetic stimulation increases and parasympathetic stimulation decreases heart rate), as well as breathing, circulation, etc., which allow the cardiovascular system to rapidly adjust to sudden physical and psychological challenges to homeostasis [1,2].

HRV indices are quantitative measures of the emergent property of interdependent regulatory systems which operate on different time scales to help our bodies to adapt to environmental and psychological challenges. The commonly used HRV metrics are quantified using temporal, spectral and nonlinear measures. Time-domain indices quantify the amount of HRV observed during monitoring periods. For example, standard deviation of all NN intervals (SDNN) is used to assess the size of the overall change in heart rate [3], and its values are different depending on the ECG recording length or segment duration; root mean square of the successive differences (RMSSD), standard deviation of differences between adjacent NN intervals (SDSD), NN50 and pNN50 are sensitive indicators of parasympathetic tone. Frequency-domain values calculate the absolute or relative amount of signal energy within component bands, i.e., ultra-low frequency (ULF), very-low-frequency (VLF), low-frequency (LF), and high-frequency (HF) bands. VLF reflects chemoreceptor regulation, thermoregulation and vasomotor activity [4]; LF is related to baroreflex regulation [5], and can reflect the regulation of both the sympathetic and parasympathetic nervous systems; HF mainly reflects the regulatory function of the parasympathetic nerve, and the index is affected by respiration. In young and healthy individuals, the HF band is significantly increased at night and decreased during the day; the LF/HF ratio can reflect the interaction and comparison of the intensity of sympathetic and parasympathetic activities [6]. The importance of nonlinear dynamic theory for HRV analysis is that it can quantitatively describe the characteristics of complex dynamic systems and extract the evolution information. The most widely used nonlinear analysis methods include detrending fluctuation analysis (DFA) [7], and entropy or multi-scale entropy based measures [8,9,10,11,12]. DFA can quantify the fractal scale index of HRV, which is suitable for detecting long-range power law correlation analysis of non-stationary time series; entropy based measures can study the complexity of HRV from single scale or multiple scale time scales [13]. Healthy biological systems exhibit spatial and temporal complexity, disease can involve either a loss or increase of variability [14]. Changes of HRV occur in several systemic diseases, such as diabetes [15], hypertension [16], heart failure [17,18], and coronary heart disease [19]. Reduced HRV is a remarkable predictor of cardiac events including sudden death in subjects with coronary artery disease, myocardial infarction and heart failure [20].

Studies have shown that HRV could be strongly influenced by environmental conditions and subject variables. [21]. For example, HRV varied with age and gender [22], ECG recording length or ECG segment duration [23], physical activity intensity [24,25], body posture change [21], et al. Besides, the intra-day and inter-day reliability of HRV measurements was also introduced in [26]. Although several studies have found an adequate reproducibility of HRV both in the time and in the frequency domain analyses [21,27,28,29,30,31], others have not made this observation [31]. Reliability and reproducibility of the various of HRV metrics obtained from short-term records is still under discussion [21,31,32,33]. Longer recording periods provide data about cardiac reactions to a greater range of environmental stimulation. For example, SDNN over 24 h will always differ to SDNN of ten minutes, and it is more accurate when calculated over 24 h than during the shorter periods; ULF usually requires 24 h Holter monitoring; VLF needs at least a recording time of 10 min or more. Therefore, guarantee of HRV reliability, stability and reproducibility is a key question in longitudinal studies or in clinical conditions, where the same data are recorded from a single individual at different times and scenarios, or in comparative studies where ECG data are recorded from different diseased and healthy control groups. Unfortunately, many HRV studies were conducted without considering such influences or had no standardized or normalized data recording criteria.

HRV analysis has become an established procedure over the past two decades [34,35] since the publication of the first guidelines [36]. There have been advances in recording technology (smaller, wearable, more mobile, more accurate devices) [37], for example, long-term RR intervals can now be measured by small chest strap and pulse watch systems [38,39]. Technology developments have decreased the costs of recording and analysis and have facilitated applications in home monitoring. With the development of wearable devices, continuous HRV metrics monitored and analyzed at patients’ home becomes feasible, which puts forward higher requirements for the standardization of environment conditions during measurement. Moreover, the importance of early detection and early treatment is self-evident, however, diseases or clinical conditions at very early stages have a less pronounced effect on HRV than severe or acute heart disease, and their alteration on HRV may be submerged in the noise of subject variables and environment conditions.

Awareness of the condition of recording and subject variables can aid interpretation of both 24-hour and short-term HRV measurements. Important factors include recording time (i.e., day or night) and recording length (i.e., 5 min, 1 h, or 24 h) [40], recording method/device [41], sampling frequency, data quality, removal of artifacts, respiration, and other environmental factors (i.e., home, school/work, hospital or laboratory) [42]. Important subject variables are age, gender, heart rate, mental stress, emotions, sleep, health status, et al. In addition, influences of body position (i.e., sitting or lying), movement, physical activity, and tasks can all affect measurements subtly or even greatly by changing autonomic nervous system activation, breathing mechanics, and emotions. The level of daily physical and mental activity should also be considered. In particular, 24-hour HRV results significantly depends on activity. Mixing HRV data of subjects with obviously different daily activities is inappropriate and may lead to values that are of little use. Many HRV related algorithms published in the literature were developed and evaluated using different public databases without checking data recording conditions. There is a lack of standardization for those HRV measurements, and thus it is difficult to make direct comparison between groups or studies.

The objectives of the present study were to investigate (1) the variability of HRV in healthy young subject under different recording conditions; and (2) subject variables in the same day of routine daily life. We mainly focus on the variability of healthy young HRV at different home monitoring scenarios in daily life, especially the routine normal daily activities which were diverse at recording time, physical activity intensity, mental activity, and body postures.

## 2. Data and Methods

### 2.1. Protocol and Data

A total of 56 volunteered healthy college students aged 21.1 ± 2.1 years (mean ± std) from Southeast University in China took part in this study, including 28 males (21.5 ± 2.4 years) and 28 females (20.8 ± 1.7 years). All participants were informed the experimental protocol and matters needing attention, then signed the written informed consent prior to participating in the study. This study was approved by IEC (Independent Ethics Committee) for clinical research of Zhongda Hospital, affiliated to Southeast University (No. 2019ZDSYLL073-P01).

ECG recordings were collected by a FDA (U.S. Food and Drug Administration) approved ambulatory electrocardiogram monitor (DynaDx Corporation, Mountainview, CA, USA) with a computer-based data-acquisition system and a sleep quality analysis software. The ECG recording equipment was a single-lead Holter device that can record ECG for over 24 h. The study was conducted at the campus of Southeast University. All participants were instructed to wear the ECG device for at least 20 h from Friday night earlier than 23:00 (before going to bed as usual) to Saturday night 19:00, and to follow the experiment protocol during the whole day. Sampling rate of ECG monitoring was set to 512 Hz.

Participants in this study were required to be healthy, free of cardiovascular diseases, diabetes, mental conditions, sleep apnea and other diseases that have a known impact on HRV, and have no adverse life behaviors, such as smoking and drinking alcohol. Female students were not in menstruation period. Subjects were also required to fill in the self-rating depression scale (SDS), profile of mood states (POMS) and Pittsburgh sleep quality index (PSQI) to avoid the influences due to mental and sleep related issues.

Of the 60 initially recruited volunteers, 56 recordings were included and 4 recordings were excluded due to low data quality.

The data collection protocol was shown in Table 1. The experiment was conducted for 20 h under various recording conditions: sleeping at night, reading quietly in different body postures, brisk walking at a certain exercise intensity, and free-running daily activities (after meals) in the morning, noon and evening.

Night sleeping: Subjects were directed to go to bed at 23:00 and wake up at 7:00 to guarantee 8-h sleep. Sleep quality and staging analysis were performed by the ‘sleephousekeeper’ software. The sleep and wake-up time were calibrated via self-reported forms filled in by the subjects. To keep a comparable ECG recording length with the other activities during daytime, the sleep ECG signals in the period of 01:00–02:00 were selected for subsequent HRV analysis.

Reading: Subjects were required to read paper books of general science in a quiet environment (i.e., in their dormitory) in specified body postures, consisting of sitting posture reading and lying posture reading. Lying posture reading included lying on the left, lying on the right and lying on the back.

Brisk walking: Subject were required to walk at a faster speed than usual on a 400 m plastic track at the campus.

Free-running daily activities (FreeDA): Subjects were asked to follow their normal daily activities except performing physical exercise or taking a shower/bath. They were free to choose whatever they want to do, such as chatting with friends, surfing the Internet, writing homework, cleaning the room, doing laundry and performing other daily activities, but the same activity cannot be run more than 15 min. ECG signals during FreeDA were recorded in three different time periods, i.e., morning, noon and evening, beginning at about 30-min after meals.

### 2.2. Data Preprocessing

The third-order band-pass Butterworth filter and zero-phase shift filter with a cut-off frequency of 2 Hz to 30 Hz were first used to eliminate the baseline drift, power frequency interference and high frequency noise, which can make the QRS complex (including the Q wave, R wave, and S wave) more prominent, and avoid changing the position of R peaks. At the same time, for those serious noises that may not be resolved using above procedure, a manual removal method was adopted, i.e., the ECG signals drowned in serious noise (probably due to unstable contact between the wearable device and the skin) were set to zero. All ECG recordings were carefully checked with ectopic beats, very few ectopic beats were found and eliminated (i.e., set to zero), and only ECG signals with normal R peaks (i.e., N peaks) were remained.

### 2.3. High-Quality RR Interval Detection and De-Noising

At present, plenty of R-peak detection methods have been proposed on ECG signal analysis. However, when applying these detectors on ECG signals collected in daily life, especially via wearable single-lead ECG devices, the R peak detection accuracies were usually unsatisfying. In this study, the ECG signals during brisk walking and free-running daily activities contains more noise than ECG collected during sleeping and reading. To compare HRV measured in different conditions, we should keep the data quality of detected RRIs at the same or similar level. A single R-peak detection method was not qualified on this database. Hence, we proposed a method combining five commonly used R-peak detection methods to get high-quality RRIs from noisy ECGs.

The five popular R-peak detection methods applied in this study were ‘Pan-Tompkins’ [43,44], ‘jqrs’ [45], ‘mteo’ [46], ‘nqrs’ [47], and ‘sixth power’ [48]. Each R-peak detection method has its own advantages. Details of the proposed method to get high-quality RRIs from noisy ECGs are as follows:

1. Detect R peaks of the ECG samples with five detection methods. Five sets of initial detection results were obtained.
2. Categorize the ECG sample by template matching and select weights by the category. There were five categories in total that corresponding to five sets of weights. A set of weights included five weights corresponding to five detection methods. For a specific kind of ECG signal, the method with better performance was assigned more weight.
3. Calculate the “scores” of each R-peak annotation in five sets of detection results in (1) based on the weights in (2). A high score indicated that the annotation might be correct.

The five sets of R detection results were merged into a vector, and the scores of the initial R-peak annotations obtained in Step (1) were calculated one by one.

First, to calculate the score of R-peak annotation j, the score was calculated based on the weight of the detection method that marked annotation j.

Then, find out whether there were other R-peak annotations marked by other four methods in the left or right range (i.e., within 50 ms around the center of annotation j) of annotation j. There would be some deviation between the results of several detection methods. When other annotations within the range were more concentrated on annotation j, it is more likely that j was a correct R-peak annotation. Therefore, the score of annotation j was inversely related to the distances from it to other annotations within the range. The score of R-peak annotation j was defined in expression (1).

(1) $$score_{j}=weight_{j}\sum_{i}\frac{fs\cdot weight_{i}}{100\cdot \left|annotation_{j}-annotation_{i}\right|},$$

where $weight_{j}$ is the weight corresponding to the detection method that marked $annotation_{j}$, $annotation_{i}$ is the annotation within the range, fs is the sampling rate of ECG signals.

Finally, a threshold was used to filter out the R-peak annotations with high scores.

(2) threshold=0.8·mean,

where mean is the average score of the five R-peak annotations before the annotation being calculated.

The parameters were adjusted according to the accuracy of the data set.

**Figure 1:**
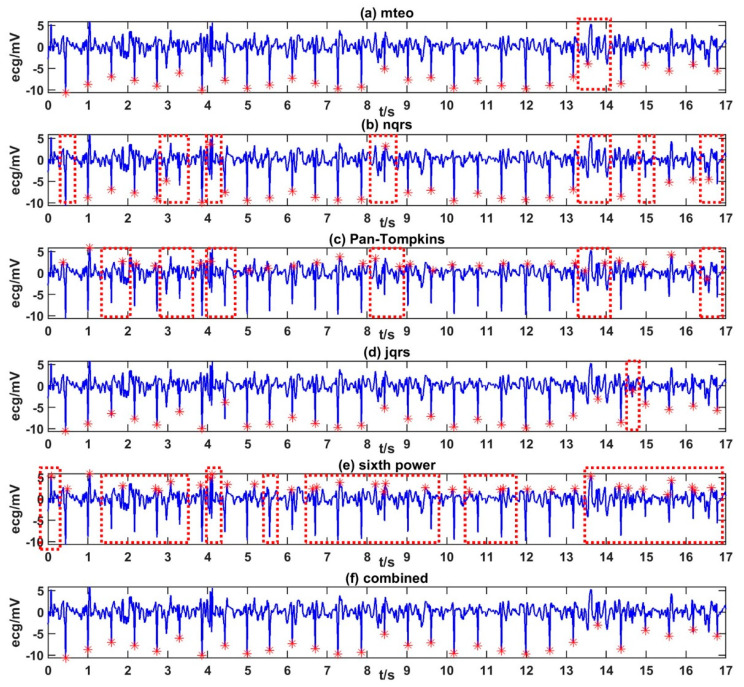
Examples of R-peak annotation results for the same electrocardiograph (ECG) segment using five popular R-peak detection methods of (**a**) meto (**b**) nqrs (**c**) Pan-Tompkins (**d**) jqrs (**e**) sixth power, and the proposed method of (**f**) combined. Missed or wrong annotations are within the red dash lines.

A more accurate R peak position were obtained. Figure 1 shows examples of the R peak labeling results.

**Figure 2:**
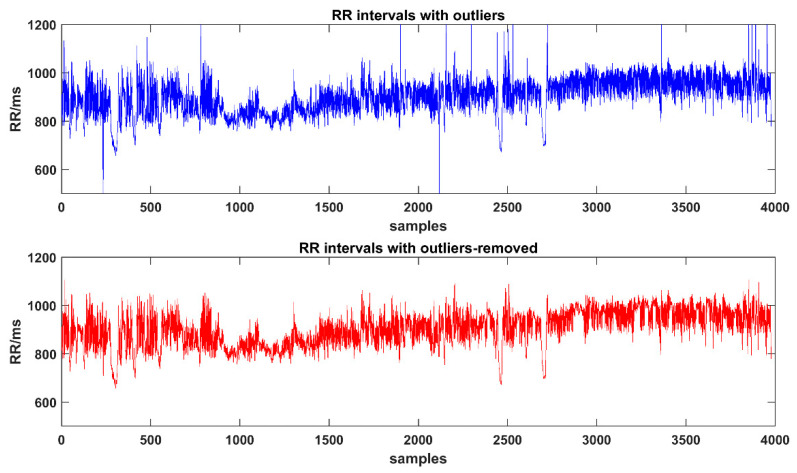
The results of using the Thompson algorithm to remove R-peak to R-peak (RR) outliers.

All detected RR time series were carefully checked with artifacts, ectopic beats or outliers. Thus, in this study, the RR intervals are the same meaning of NN intervals. Outliers would have a certain impact on the results of HRV analysis. Here we chose the Thompson algorithm [49] to find outliers in the RRIs, which divided the original sequence into small windows according to the customized length to find out outliers and then to replace them with the average of adjacent points. In this study, four windows with lengths of 15, 50, 100, and 200 data points were selected to remove outliers. Figure 2 shows the results of using Thompson algorithm to eliminate outliers.

### 2.4. HRV Metrics

In this study, we evaluated the variability of healthy HRV mainly based on the widely-used HRV linear metrics (i.e., time-domain and frequency-domain) and nonlinear metrics. All HRV metrics were analyzed under nine different recording scenarios, including sleeping at night, sitting (reading), lying down (reading) separately on the left side, right side and the back, brisk walking and free-running daily activities separately in the early morning, noon and evening. All indices and their definitions analyzed in this study are shown in Table 2. We used the PhysioNet Cardiovascular Signal Toolbox [50] to perform indicator calculations, except for fuzzy entropy and multi-scale fuzzy entropy (MATLAB codes from Hamed Azami and Javier Escudero Rodriguez [51,52]) and permutation entropy and multi-scale permutation entropy (MATLAB codes provided by Gaoxiang Ouyang [53,54]).

#### 2.4.1. Linear HRV Metrics

Linear HRV metrics include time-domain indices and frequency domain indices. Time-domain indices quantify the amount of variability in measurements of the time period between successive heartbeats. For example, SDNN is used to assess the size of the overall change in heart rate, while SDANN relates to the variance below the 0.0033 Hz spectral frequency. RMSSD, SDSD, NN50 and pNN50 reflect the beat-by-beat changes in heart rate, that is, the size of the short-term changes in heart rate.

Frequency-domain measurements estimate the distribution of absolute or relative power at four frequency bands. The Task Force of the European Society of Cardiology and the North American Society of Pacing and Electrophysiology (1996) divided heart rate oscillations into ULF, VLF, LF, and HF bands (see Table 2). ULF usually requires long-term recordings of Holter monitoring, and thus was not included in this study.

#### 2.4.2. Nonlinear HRV Metrics

Heartbeat is a chaotic dynamic process. The complex interactions between electrophysiology, hemodynamics, autonomic nervous system, fluid regulation and other systems lead to nonlinear phenomena. The oscillations of a healthy heart are complex and nonlinear. The variability of heart rate provides the flexibility to rapidly cope with an uncertain and changing environment. Thus, the traditional linear HRV metrics are not enough to characterize the complex dynamics of generation of the heartbeat. Nonlinear analysis methods differ from the conventional linear HRV methods because they do not assess the magnitude of variability but rather the unpredictability, fractability and complexity of RRIs (or IBIs) time series, which results from the complexity of the mechanisms that regulate HRV. In this case, the nonlinear method is of great value in analyzing the RRIs time series.

This section introduces approximate entropy (ApEn) [8], fuzzy entropy (FuzzyEn) [9,51], sample entropy (SampEn) [10], permutation entropy (PeEn) [11,53], detrending fluctuation analysis (DFA, alpha1 and alpha2) [7], multi-scale entropy (MSE) [12], multi-scale fuzzy entropy (MFE) and multi-scale permutation entropy (MPE). Such metrics have potentially important applications with respect to evaluating both dynamical models of biologic control systems and bedside diagnostics. For example, a wide class of disease states, as well as aging, appear to degrade physiologic information content and reduce the adaptive capacity of the individual. Loss of complexity, therefore, has been proposed as a generic feature of pathologic dynamics.

- **Approximate Entropy (ApEn)**

ApEn is a conventional measure to quantify the irregularity or complexity of time series. It reflects the probability of new subsequences, which is a conditional probability method to measure the possibility of new information in time series. Therefore, the more complex time series corresponds to the larger ApEn.

1. For a given time sequence u(i):1≤i≤N and given *r* and *m*, reconstruct to obtain (*N* − *m* + 1) subsequences X(i)=u(i),u(i+1),u(i+2),…,u(i+m−1).
2. Calculate the distanced $d_{m}\left[X\left(i\right),X\left(j\right)\right]$ between any two reconstructed subsequences. The distance is determined by the maximum difference between the corresponding position elements of the two sequences, including the distance when i=j.
3. Calculate the ratio $C_{i}^{m}\left(r\right)$ of the number of distances less than r:
  (3) $$C_{i}^{m}\left(r\right)=\frac{num\left(d_{m}\left(X\left(i\right),X\left(j\right)\right)<r\right)}{N-m+1},$$
4. Calculate the average similarity rates $\Phi_{m}\left(r\right)$ and $\Phi_{m+1}\left(r\right)$ when the number of subsequences are m and m+1:
  (4) $$\Phi_{m}\left(r\right)=\frac{\sum_{i=1}^{N-m+1}\log\left(C_{i}^{m}\left(r\right)\right)}{N-m+1},$$
5. Approximate entropy:
  (5) $$ApEn=\Phi_{m}\left(r\right)-\Phi_{m+1}\left(r\right),$$

- **Sample Entropy (SampEn)**

SampEn is an improved complexity measurement based on the concept of ApEn. The initial calculation process is the same as ApEn.

SampEn is calculated as:

(6) $$SampEn=-\log\left[\frac{\Phi_{m+1}\left(r\right)}{\Phi_{m}\left(r\right)}\right],$$

- **Fuzzy Entropy (FuzzyEn)**

FuzzyEn is improved on the basis of SampEn, which describes the probability of a new pattern in a complex system containing noise. It is the entropy of a fuzzy set, which loosely represents the information of uncertainty.

1. Different from step a) in ApEn, get $X\left(i\right)=\left[u\left(i\right),u\left(i+1\right),u\left(i+2\right),\ldots ,u\left(i+m-1\right)-u_{0}\left(i\right)\right]$.
  (7) $$u_{0}\left(i\right)=\frac{1}{m}\sum_{j=0}^{m-1}u\left(i+j\right),$$
2. Calculate the distance $d_{ij}^{m}\left[X\left(i\right),X\left(j\right)\right]$ (i≠j) between any two reconstructed subsequences.
3. Introduce fuzzy membership function $A_{ij}^{m}\left(r\right)$ and calculate the average $C_{i}^{m}\left(r\right)$ for each i:
  (8) $$A_{ij}^{m}\left(r\right)=e^{-{\left(\frac{d_{ij}^{m}}{r}\right)}^{2}},\;C_{i}^{m}\left(r\right)=\frac{\sum_{i=1,j\neq i}^{N-m+1}A_{ij}^{m}\left(r\right)}{N-m},$$
4. Calculate the average similarity rates $\Phi_{m}\left(r\right)$ and $\Phi_{m+1}\left(r\right)$ when the number of subsequences are m and m+1:
  (9) $$\Phi_{m}\left(r\right)=\frac{\sum_{i=1}^{N\;-m+1}C_{i}^{m}\left(r\right)}{N-m+1},$$
5. Fuzzy entropy:
  (10) $$FuzzyEn=-\log\left[\frac{\Phi_{m+1}\left(r\right)}{\Phi_{m}\left(r\right)}\right],$$

- **Permutation Entropy (PeEn)**
- PeEn is similar to ApEn, SampEn and FuzzyEn, which are all used to measure the complexity of time series. PeEn introduces the idea of permutation when calculating the complexity between reconstructed subsequences.

1. Consistent with step a) in ApEn, get X(i).
2. Incremental sorting is performed inside each X(i) and mapped to m! kinds of permutations. The internally sorted order of X forms a symbol sequence Y(i), and its probability distribution is represented by P(i).
3. Permutation entropy:
  (11) $$PeEn=-\frac{\sum_{i=1}^{N--m+1}P\left(i\right)\log\left(P\left(i\right)\right)}{\ln\left(m!\right)},$$

- **Detrended Fluctuation Analysis (DFA)**

DFA is a scaling method commonly used for detecting long-range correlations in non-stationary time series. It has two important advantages over other scaling analysis: it reduces noise effects and removes local trends, it is relatively unaffected by any non-stationarities.

In the standard DFA, basically, a time series is integrated to obtain a type of random-walk profile. Then, this sequence is divided into non-overlapping boxes of equal size n. In each box, the local trend is estimated by an m-degree polynomial fitting. In a subsequent step, the root-mean-square fluctuation, denoted by $F_{m}\left(n\right)$, of the difference between the integrated sequence and the polynomial fits is calculated for each n. Finally, assuming that such fluctuations meet a power-law with respect to the box size, a scaling factor α is computed as the slope of the following fluctuation plot.

(12) $$ℱ\overset{\mathrm{def}}{=}\left\{log\left(n\right)\;versus\;log\left(F_{m}\left(n\right)\right)\right\},$$

- **Multi-Scale Entropy (MSE)**

Disease and aging will reduce the physiological information and the adaptability of individuals, and the complexity of physiological indicators will be reduced. However, some conditions are associated with highly unstable fluctuations, such as arrhythmias, and the increase in single-scale entropy of such pathological signals contradicts the common perception that the complexity of physiological indicators decreases under pathological conditions. To solve this problem, MSE is proposed.

MSE analyses the complexity of time series from multiple time scales. The algorithm consists of three steps:

1. Time series coarse graining: for the given time series u(i):1≤i≤N, construct a continuous coarse-grained time series *y^τ^*:
  (13) $$y^{\tau }\left(j\right)=\frac{1}{\tau }\sum_{i=\left(j-1\right)\tau +1}^{j\tau }u\left(i\right),1\leq j\leq N/\tau ,$$
  where τ is the scale factor, as τ = 1, 2, 3...
2. Calculate the SampEn of each coarse-grained time series *y^τ^*.
3. Draw the graph line with the scale factor τ as the independent variable and the SampEn of the corresponding scale sequence as the dependent variable.

Similarly, multi-scale fuzzy entropy (MFE) and multi-scale permutation entropy (MPE) were calculated.

**Figure 3:**
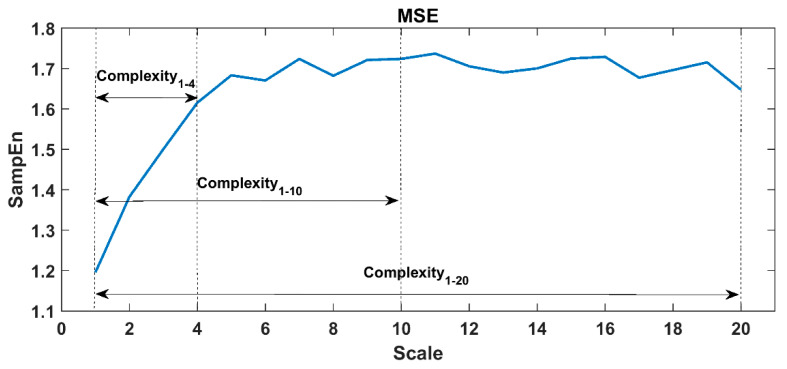
Multi-scale entropy (MSE) analysis results of a set of RR interval.

When calculating ApEn, SampEn and FuzzyEn, the embedding dimension m was set to 2, and the similar tolerance r was set to 0.15 times the standard deviation of the sequence. In PeEn, the embedding dimension was set to 3. In the DFA analysis, the fractal interval was set to 4–64, alpha 1 corresponded to 4–15, and alpha 2 corresponded to 16–64. In this study protocol, each task proceeded for 30 to 60 min, thus the scale factor τ in MSE/MFE/MPE was set to 1~20 for 60-min tasks and 1~10 for 30-min tasks (i.e., lying reading tasks) to ensure the reliability of large-scale analysis. A representative MSE curve of the 60-min RRIs time series was shown in Figure 3, here we constructed 20 coarse-grained time series from the original RRIs. The complexity indices were defined by calculating the area under the MSE curve (see definitions in Table 2), i.e., Complexity_1-20_ (area under the whole MSE curve), Complexity_1-10_ (area under the scales 1-10), and Complexity_1-4_ (area under the small scales 1–4, which mainly demonstrated the influence of breathing or vagal tone).

### 2.5. Statistical Analysis

IBM SPSS Statistics 21.0 was used for statistical analysis in this study. The main purpose of statistical analysis is to determine whether there are significant differences between specific groups.

One-sample K-S hypothesis test was used to test whether it follows a normal distribution (significance level α = 0.05). For the population followed a normal distribution, one-way ANOVA test was performed on multiple sets of control samples and independent sample t-test was performed for the analysis between the two sets of samples. For the population did not follow a normal distribution, non-parametric tests were performed. The Mann–Whitney U test was used for the comparison of the two sets of samples, and the Kruskal–Wails H test was used for multiple samples. The significant difference was defined as the *p*-value < 0.05.

## 3. Results

We firstly observed the 20-h circadian rhythm of the real-time RR intervals in healthy young college subjects. Then, the variability of healthy HRV metrics was presented from the following five different categories: (1) different routine activities in daily life (free-running daily activities, reading, sleeping and brisk walking); (2) different recording time period in the same day during free-running daily activities (morning, noon and evening); different body position of (3) sitting and lying, and (4) lying on the left side, right side and supine; (5) gender influence (males and females). Finally, we selected SDNN, LF-HF-ratio, and Complexity_1-10_ as representative results to show the overall variation of healthy HRV in the nine different states within the 20-h recording period.

### 3.1. Temporal Fluctuations and 20-Hour Trend of RR Intervals

**Figure 4:**
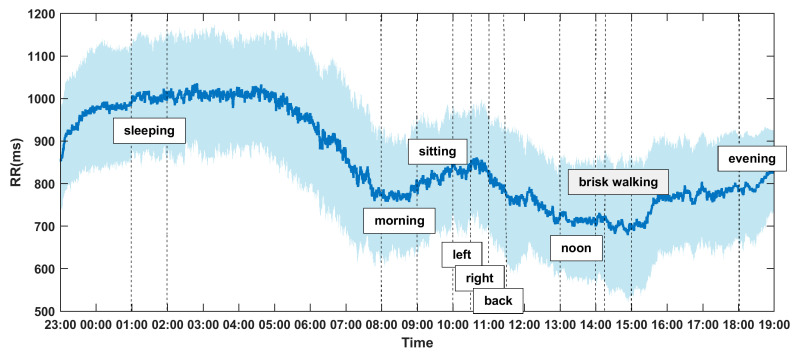
The real time dynamic fluctuations and overall trend of RR intervals (RRIs). The RRIs data segments analyzed in this study were marked within dash lines.

The 20-h dynamic fluctuations and trend of RR intervals (inverse of instantaneous heart rates) affected by circadian rhythm and protocol tasks could be observed in Figure 4. The shaded part represents the standard deviation, and the curve in the middle represents the mean RR intervals of 56 healthy volunteers. It can be observed that the RR values are higher and more stable from 23:00–7:00 (night sleeping, lower heat rate), and dramatically decreased immediately after waking up in the morning with a visible local nadir, which indicates the heart rate morning peak. The RR intervals dropped into another nadir during brisk walking at around 14:15–15:00. From 9:00–11:30 (reading quietly), the heart rate slows down, and RR reaches a small peak, but it is still lower than the sleep state. The observed overall fluctuations and trends are consistent with existing theory and common sense, which proves that the recorded ECG data and extracted RR time series are reliable.

### 3.2. Variability of HRV under Different States in Daily Life

To evaluate the variability of HRV in different daily scenarios, four different routine daily activities were considered, including FreeDA, reading, sleeping and brisk walking. In this section, the HRV results of FreeDA for each subject were obtained by taking the averaged value of HRV metrics in the morning, noon and evening for this subject, and the reading state HRV was analyzed only while sitting.

Comparison results of HRV metrics under the four different daily activities were shown in Table 3:

- Significant differences were observed for all HRV metrics among the four different states in daily life. Some of the HRV metrics (i.e., RR-mean, RR-median, SampEn, FuzzyEn and Complexity_1-20_) existed significant difference between any two states. For the other HRV metrics, significant differences were found between most two daily life states.
- For RR-mean, RR-median, RMSSD, SDSD, NN50, pNN50, TP, HF, HF-percentage, DFA-alpha2, SampleEn, and FuzzyEn, sleep state had the highest values, followed by reading, FreeDA, and brisk walking had the lowest values, which is sleeping > reading > FreeDA > brisk walking. The inversed trend was found in LF-percent, LF-HF-ratio and DFA-alpha1.
- ApEn and PeEn had similar trend, which is brisk walking > sleeping > FreeDA > reading.
- For the frequency-domain metrics, like VLF and LF, we got reading > sleeping > FreeDA > brisk walking. LF-percent also gave the lowest values during sleeping, but very close values during the other three activities.
- Interestingly, MSE measured Complexity_1-4_ and Complexity_1-20_ gave the highest values during reading and lowest values during brisk walking.

**Figure 5:**
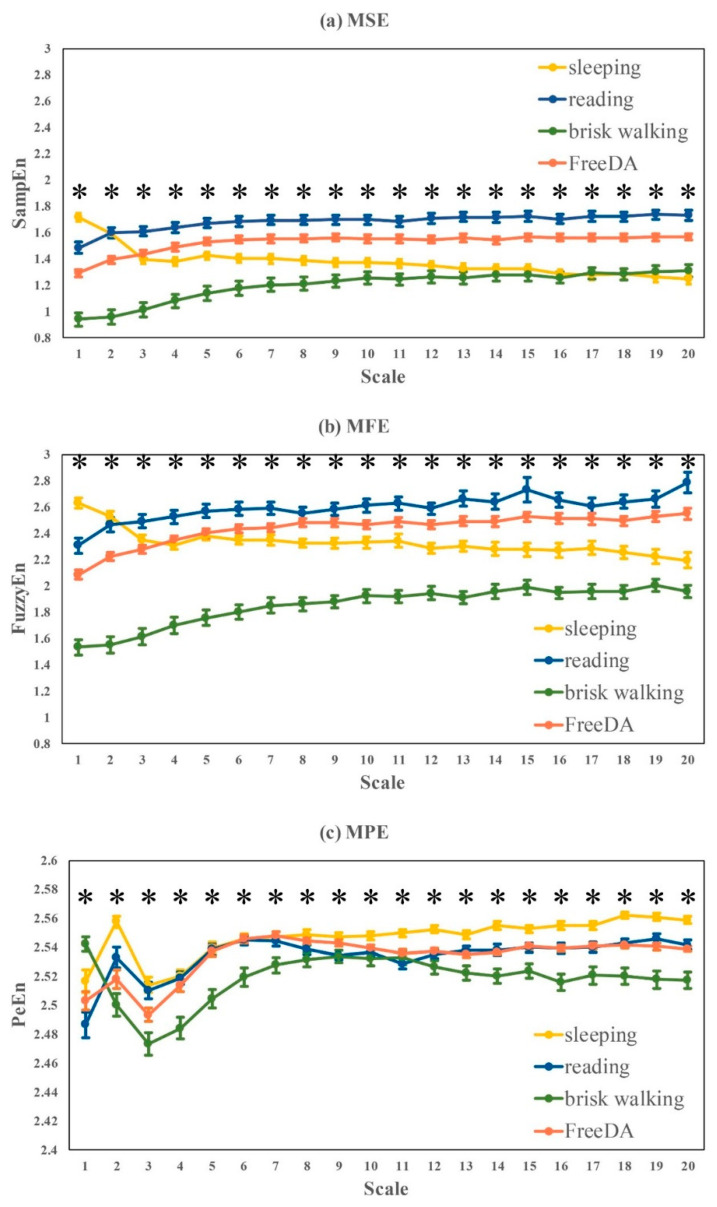
Multi-scale based entropy analysis of RR intervals recorded in different states of daily life. Curves represent mean ± standard error of each subject within a group. (* *p* < 0.05)

The comparative multi-scale based entropy curves measured by MSE, MFE and MPE was plotted in Figure 5:

- Significant differences were found among four different daily scenarios at all scales (1–20) in MSE (Figure 5a), MFE (Figure 5b) and MPE (Figure 5c) curves.
- The overall trend of MSE and MFE curves were relatively close, and the entropy values during sleep shows a downward trend as the scales increase, while the other three daytime states slowly rise on the small scales (1–5) and then towards stability on large scales (6–20) showing reading > FreeDA > sleeping > brisk walking, which is consistent with Complexity_1-20_ shown in Table 3.
- The trend of MPE curve was quite different from MSE and MFE. The entropy values at small scales (1–10) were unstable for different activities, while it could be found that sleeping > reading ≈ FreeDA > brisk walking at large scales (11–20).

### 3.3. HRV Metrics Varied with Recording Time in the Same Day during Free-Running Daily Activities

**Figure 6:**
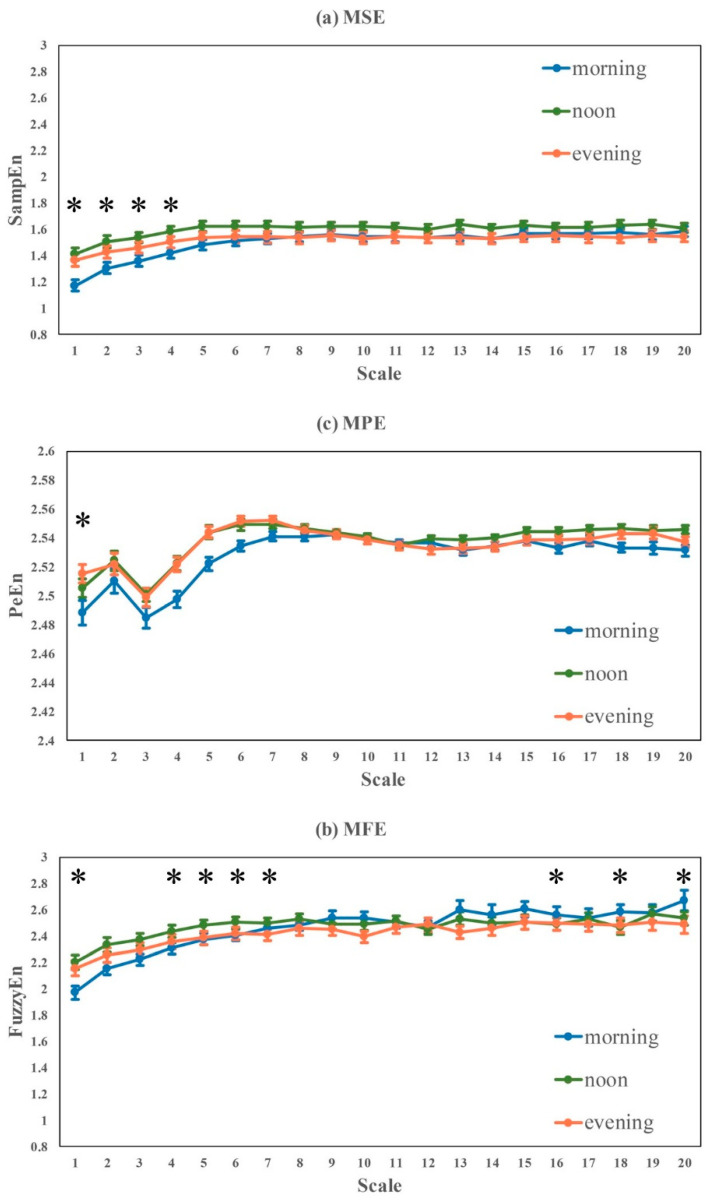
Multi-scale based entropy analysis of RR intervals recorded in in different time periods during free-running daily activities. Curves represent mean ± standard error of each subject within a group. (* *p* < 0.05)

RR interval time series obtained from subjects while they were doing one-hour free-running daily activities separately in the morning, noon and evening of the same day were included in this section. Comparison results of HRV metrics in the three time period were presented in Table 4 and Figure 6.

- No significant difference was found in all of linear HRV metrics (time- and frequency-domain), as well as nonlinear HRV metrics like DFA-alpha1, DFA-alpha2, ApEn and Complexity_1-20_.
- Significant differences were found in nonlinear HRV metrics like SampEn, FuzzyEn, PeEn and Complexity_1-4_, consistently showing entropy-based complexity: noon > evening > morning. The statistical significances were mainly from morning vs noon and morning vs evening, and no significant difference was found between noon and evening. A similar trend was found in Complexity_1-20_, but the group difference was not statistically significant.
- In Figure 6, the overall trends of the three sets of MSE, MFE and MPE curves were relatively close. The difference among MSE curves was more obvious, the entropy values at small scales (1–4) measured in the morning is significantly lower than that at noon and in the evening. The MSE curve measured at noon is the highest, but the difference was not statistically significant at large scales. Significant differences were found in MPE at some specific scales, e.g., scales 1, 4–7, 16, 18 and 20.

### 3.4. Variability of HRV Metrics Influenced by Body Postures

#### 3.4.1. Sitting and Lying

**Figure 7:**
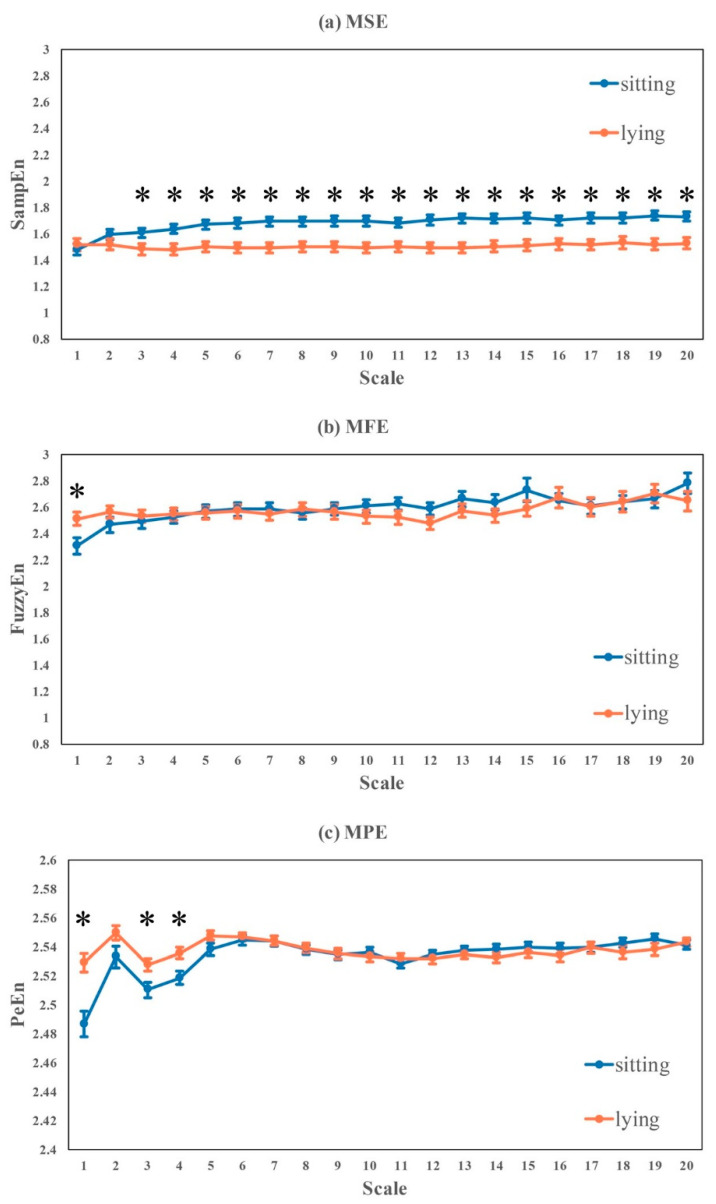
Multi-scale based entropy analysis of RR intervals recorded while sitting and lying postures. Curves represent mean ± standard error of each subject within a group (* *p* < 0.05).

Subjects were asked to sit and then lie down while reading general science books quietly. Lying posture consisted of lying on the left, right and back. HRV metrics analyzed for sitting and lying were presented in Table 5 and Figure 7:

- Significant differences were found in linear HRV metrics (time- and frequency-domain) like RR-mean, RR-median, SDNN, SDANN, NN50, pNN50, TP, VLF, LF, HF, LF-HF-ratio, LF-percent and HF-percent, consistently showing lying > sitting, except LF-percent and HF-percent where sitting > lying. RMSSD and SDSD also had similar trend of lying > sitting, but the differences were not statistically significant.
- No significant difference was found in nonlinear HRV metrics like DFA-alpha1, DFA-alpha2, SampEn and Complexity_1-4_.
- Significant differences were found in ApEn, FuzzyEn, PeEn and Complexity_1-10_, where FuzzyEn and PeEn showed lying > sitting, and ApEn and Complexity_1-10_ showed sitting > lying.
- For the multi-scale based entropy curves in Figure 7, MSE, MFE and MPE performed uniquely. Complexity of RR intervals while sitting was significantly higher than lying posture at large scales (3–10) in MSE, which was inversed in MFE at scale 1 and MPE at scales 1, 3 and 4.

#### 3.4.2. Lying in Different Body Postures

**Figure 8:**
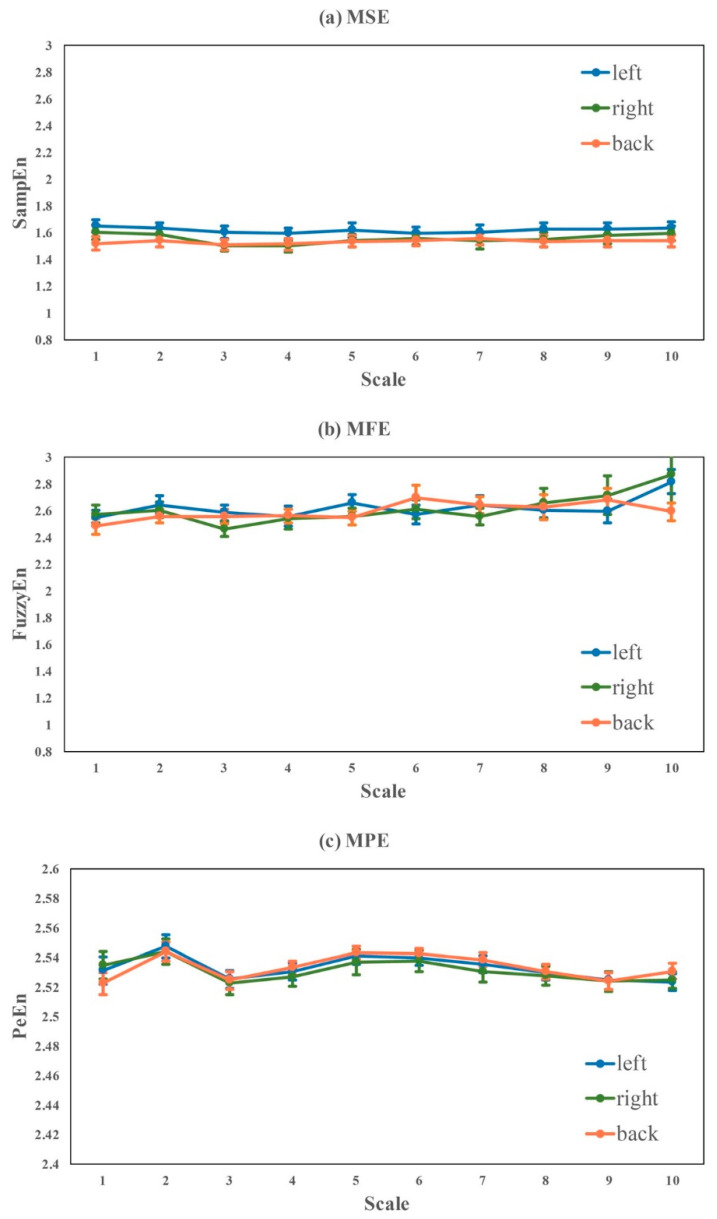
Multi-scale based Entropy analysis of RR intervals recorded while different lying postures. Curves represent mean ± standard error of each subject within a group.

Here, influence of three different lying postures (i.e., lying on the left, lying on the right and lying on the back) were evaluated in Table 6 and Figure 8. We found that, except for num-rr, there was no significant difference in any of the HRV metrics among different lying postures. However, there was the same trend of right > back > left in most of linear HRV measures like SDNN, RMSSD, SDSD, NN50, VLF, LF and HF. Interestingly, the nonlinear HRV measures of SampEn, FuzzyEn, PeEn, Complexity_1-4_ and Complexity_1-10_ indicated another trend that the lowest complexity appeared while lying on the back.

### 3.5. Gender Influence

In this section, gender influence was evaluated under the involved seven states or scenarios for all the analyzed HRV metrics. Results were shown in Table 7.

- Generally, males have significant bigger inter-beat RR intervals (i.e., slower heart rate) than females under all seven scenarios (see RR-mean and RR-median in Table 7).
- For time-domain metrics, males had significant higher SDNN during sleeping, sitting (reading), FreeDA in the morning and evening; other time-domain metrics was not influenced much by gender, e.g., significant differences were found in RMSSD, SDSD and NN50 only during FreeDA in the evening, and in SDANN only during sleeping at night; males had significant higher pNN50 than females during sitting and FreeDA in the evening.
- For frequency-domain metrics, males had significant higher TP, VLF and LF in most of scenarios except brisk walking, sleeping (VLF and LF) and lying (TP and LF), and had higher LF-HF-ratio during sleeping, lying and FreeDA at noon; other frequency metrics was not influenced much by gender, e.g., males had significant higher HF only during sitting, lower HF-percentage only during lying, and no significant difference was found in LF-percent.
- For nonlinear metrics, males had significant higher DFA-alpha1 during lying and FreeDA at noon, higher DFA-alpha2 during sleeping, and lower ApEn during brisk walking and FreeDA in the morning, noon and evening; other entropy measures were not influenced much by gender, e.g., males had significant lower SampEn only during lying, and higher (borderline) FuzzyEn only during brisk walking; significant differences were found in PeEn during sleeping and brisk walking; no significant differences were found in MSE measured complexity metrics.

### 3.6. The Variability of MSE during the Whole Study Protocol

**Figure 9:**
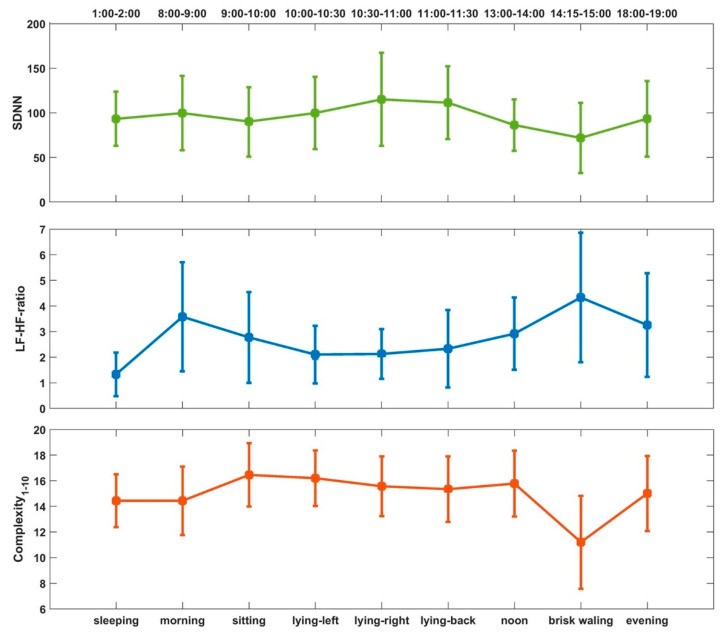
Overall variance of standard deviation of RR intervals (SDNN), low-frequency (LF) to high-frequency (HF) ratio (LF-HF-ratio), and complexity.

To present the overall changes of HRV during the whole study protocol, we selected three conventional measures from each category of HRV metrics, e.g., SDNN in time-domain, LF-HF-ratio in frequency-domain, and MSE based complexity in nonlinear analysis. Figure 9 shows the mean and standard deviation of SDNN, LF-HF-ratio and Complexity_1-10_ for all subjects.

## 4. Discussion

In this study, we focused our investigation on the variability of HRV influenced by data recording conditions for healthy young subjects that have been frequently used as healthy controls in HRV studies, and we did not study the influence of aging and disease. By grouping the HRV metrics into five different categories, we found the following: (1) HRV metrics for healthy subjects, both linear measures and nonlinear measures, were significantly influenced by routine life states and physical activities, ECG recording time (morning, noon, evening), body postures, and gender. (2) Although significant differences between sitting and lying were observed, no significant difference was found among different lying positions. (3) Nonlinear measures were more sensitive to ECG recording time than time- and frequency-measures. Thus, these findings demonstrated that the standardization of ECG data collection and HRV analysis should be emphasized in HRV related studies.

Our findings are consistent with common sense and previously published results. The cardiovascular system shows obvious daily rhythms in most physiologic parameters including HR and blood pressure [55]. Generally, HRV parameters tend to increase during the night and decrease during the day, but when HR suddenly changes from a low point at night to a higher daily value, the variability around arousal is greater [56,57]. The lowest HRV in the morning [58] corresponds to the period of the highest occurrence of ventricular tachycardia and sudden death [59,60,61]. When doing more intense exercises (like brisk walking), compared with more relaxed daily activities, the sympathetic nerve activity is stronger, the parasympathetic nerve activity is weak, the complexity of HRV is lower, and the body’s ability to adapt to environmental changes will also be weakened. In different time periods, the HRV changes less, but the complexity of the morning was significantly lower than that of noon and evening. It may reflect that the morning is a more stressful state, which is an important time for the body to wake up. The highest complexity is obtained at noon, which may be related to biological rhythm and temperature. We also found that the influence of posture on HRV is greater than that of different periods of the day. Sitting and lying postures showed obvious differences in sympathetic and parasympathetic excitability and complexity. However, we didn’t find significant difference in HRV between lying postures of left, right and supine positions. Similarly, studies have shown that, from supine to standing, HR increased significantly, time-domain HRV metrics decreased correspondingly. Frequency domain variables decreased significantly from supine to sitting position [62].

We presented the MSE measured complexity, instead of MFE and MPE, in the Table 2, Table 3, Table 4, Table 5, Table 6 and Table 7 and its overall changes during the seven scenarios within 20-h in Figure 9. The multi-scale based entropy analysis was firstly proposed by Costa et al. [12] using sample entropy, and named it MSE. MSE is the original and also the most widely used multi-scale complexity measure in physiological signals, such as RR intervals. For the past two decades, hundreds of research studies have applied MSE to investigate problems ranging from heart disease, diabetes, traumatic brain injury, frailty in the elderly, to neurological diseases, which demonstrated that the complexity of healthy physiologic systems is higher than those with advanced aging or pathology [12]. Hence, it is important to know how to define the baseline of ‘healthy MSE’ based on the observed variability influenced by data recording conditions.

Entropy curves of MFE and MPE are more stable than MSE during different life states or postures. MFE and MPE are more applicable for time series with large noise interference, so they were less influenced by the noises generated from different recording conditions, and thus didn’t show as much variations as MSE in this study. On the other hand, small fluctuations caused by minor health problems could not be quantified by MFE or MPE, either. MSE is more sensitive to RR time series fluctuations than MFE and MPE, and even minor changes in complexity could be monitored by MSE, which is more suitable for the detection of HRV alteration caused by disease in its early-stage. Under these conditions, to apply MSE, we should set up the same measuring protocol for all the involved groups for easy comparison.

In addition, current R peak or P peak detection algorithms generally have strict requirements for ECG or PPG signal quality. It is expected that new algorithms with better noise resistance could be proposed to extract high quality RR or PP intervals from data collected in daily life or during physical activities, which is of great significance to apply HRV for daily health monitoring and management.

In conclusion, the main contribution and new insights of this presented study could be summarized as follows: (1) our findings could provide supportive evidence for future HRV measurement during home monitoring because all the considered variables were controlled properly, e.g., ECG signals of different life states for each subject were recorded within the same day, only healthy young subjects were involved and their ECG recording time periods and environments were almost the same, age and gender were controlled, et al.; (2) instead of recording length, this study focus more on the influence of ECG recording time to HRV, and found the lowest heart rate complexity in the morning and highest complexity at noon, emphasizing the special role of morning in a day; (3) more detailed body postures (i.e., lying-left, lying-right, lying-back) were studied in addition to HRV comparison between sitting and lying; (4) more importantly, various of entropy and multi-scale entropy based measures were analyzed in this study, compared with traditional linear HRV metrics and nonlinear DFA indices, entropy and multi-scale entropy based measures were more sensitive to HRV variations, especially caused by ECG recording time, at recording length of 30–60 min, demonstrating that entropy based measures should be more helpful to detect the sub-healthy status or early-stage diseases that may result in minor variations from healthy HRV, especially during early morning.

## 5. Study Limitations and Future Directions

The presented study is a pilot study with 56 subjects, and thus the results cannot serve as the HRV norms. All participants were healthy young college students who do not represent the whole spectrum of healthy young people. Although we chose to collect data during weekend to reduce the influences of specific course learning during weekday, there are still diversities between college students and other young people.

To evaluate the influence of body postures, subjects were asked to read books (topic of general science) instead of resting (doing nothing). The protocol for recording ECG signals during different body postures lasted for 2.5 h. It is challenging to have the participants to maintain stable resting state for such a long period, thus reading book was used as an alternative protocol.

Gender-related differences were found for some HRV metrics under specific conditions in this study, and were not found for other metrics. Due to small sample size (with equal number for males and females), we didn’t evaluate group differences by gender in Table 3, Table 4, Table 5 and Table 6.

The ECG signals recorded in the period of 01:00–02:00 were picked up to represent sleeping state. According to sleep staging report, during this period, most subjects were in deep sleep, some subjects were in light sleep, and nobody was awake. The effects of different sleep stages on HRV should be further investigated in future studies, for example, we are analyzing HRV changes in different sleep stages in another ongoing study on 3–14 years children.

Due to limit of data acquisition time and financial support, other important factors have not been covered in this study, such as age, temperature and season, recording time length, mental stress, emotion, sampling rate, and so on. Further studies are needed to explore those interesting factors.

## Figures and Tables

**Table 1:** Data collection protocol: activities and its corresponding duration.

Activity			Start and End Time	Duration (min)
Night Sleeping			1:00–2:00	60
Body postures (Reading)	Sitting		9:00–10:00	60
	Lying	Left	10:00–10:30	30
		Right	10:30–11:00	30
		Back	11:00–11:30	30
Brisk Walking			14:15–15:00	45
Free-running daily activities	Morning		8:00–9:00	60
	Noon		13:00–14:00	60
	Evening		18:00–19:00	60

**Table 2:** Heart rate variability (HRV) metrics analyzed in this study.

Parameter (Unit)	Description
**Linear Metrics**	
Time-domain	
Num-RR	The number of RR intervals analyzed in HRV metrics
RR-mean (ms)	Mean of RR intervals
RR-median (ms)	Median of RR intervals
SDNN (ms)	Standard deviation of RR intervals
RMSSD (ms)	Root mean square of successive RR interval differences
SDSD (ms)	Standard deviation of successive RR interval differences
SDANN (ms)	The standard deviation of the average value every 5 min in the RR interval
NN50	The number of successive RR intervals that differ by more than 50 ms
pNN50 (%)	Percentage of successive RR intervals that differ by more than 50 ms
Frequency-domain	
TP (ms2)	Absolute power of total frequency band (≤0.4 Hz)
VLF (ms2)	Absolute power of the very-low-frequency band (0.003–0.04 Hz)
LF (ms2)	Absolute power of the low-frequency band (0.04–0.15 Hz)
HF (ms2)	Absolute power of the high-frequency band (0.15–0.4 Hz)
LF-percent (%)	Percentage of LF in VLF, LF and HF power
HF-percent (%)	Percentage of HF in VLF, LF and HF power
LF-HF-ratio	Ratio of LF-to-HF power
**Nonlinear Metrics**	
DFA-alpha1	Short-term correlation (fractal interval 4–15) in detrended fluctuation analysis
DFA-alpha2	Long-term correlation (fractal interval 16–64) in detrended fluctuation analysis
ApEn	Approximate entropy
SampEn	Sample entropy
FuzzyEn	Fuzzy entropy
PeEn	Permutation entropy
MSE	Multi-scale entropy
MFE	Multi-scale fuzzy entropy
MPE	Multi-scale permutation entropy
Complexity_1–4_	Sum of sample entropy for 1–4 scales of MSE
Complexity_1–10_	Sum of sample entropy for 1–10 scales of MSE
Complexity_1–20_	Sum of sample entropy for 1–20 scales of MSE

**Table 3:** Variability of HRV under different states of daily life (data was presented as mean ± standard deviation). * *p* < 0.05.

	Sleep (n = 52)	Read-Sit (n = 51)	Walk-Brisk (n = 53)	FreeDA (n = 46)	*p*
**Num-rr**	3484.04 ± 556.64	4602.16 ± 601.85	4224.68 ± 855.01	4933.96 ± 476.43	0.000 *
**RR-mean**	1011.59 ± 134.45	774.62 ± 97.27	580.16 ± 90.77	724.41 ± 59.96	0.000 *
**RR-median**	1021.11 ± 141.76	779.89 ± 99.18	563.76 ± 93.68	727.49 ± 62.42	0.000 *
**SDNN**	93.37 ± 30.33	90.29 ± 38.44	71.95 ± 39.38	94.62 ± 28.81	0.001 *
**RMSSD**	69.25 ± 29.34	55.88 ± 56.97	25.31 ± 19.77	44.82 ± 23.61	0.000 *
**SDSD**	69.26 ± 29.35	55.88 ± 56.97	25.32 ± 19.77	44.82 ± 23.61	0.000 *
**SDANN**	45.83 ± 23.07	44.10 ± 21.24	56.75 ± 40.11	62.31 ± 24.10	0.001 *
**NN50**	1342.75 ± 598.85	962.53 ± 659.97	221.25 ± 260.81	798.13 ± 421.37	0.000 *
**pNN50**	40.09 ± 19.64	22.09 ± 15.76	5.84 ± 7.33	16.34 ± 8.76	0.000 *
**TP**	1,665,810.63 ± 449,877.49	989,060.80 ± 343,791.86	666,929.99 ± 309,226.21	870,813.42 ± 206,973.84	0.000 *
**VLF**	11,723.92 ± 9810.59	11,827.35 ± 10,470.17	4898.31 ± 8354.71	9386.96 ± 4766.43	0.000 *
**LF**	4812.40 ± 4022.08	5393.98 ± 5207.24	2360.54 ± 5377.76	4248.50 ± 2516.23	0.004 *
**HF**	4585.07 ± 3643.85	2393.98 ± 2402.09	630.69 ± 909.49	1730.02 ± 1257.34	0.000 *
**LF-HF-ratio**	1.33 ± 0.85	2.77 ± 1.13	4.33 ± 0.97	3.28 ± 1.51	0.000 *
**LF- percent**	32.13 ± 1.31	33.34 ± 1.12	33.92 ± 1.35	33.61 ± 0.84	0.000 *
**HF- percent**	31.99 ± 2.15	29.87 ± 1.63	27.13 ± 2.78	29.23 ± 1.17	0.000 *
**DFA-alpha1**	0.93 ± 0.21	1.12 ± 0.21	1.24 ± 0.18	1.17 ± 0.13	0.000 *
**DFA-alpha2**	1.12 ± 0.09	1.05 ± 0.10	1.04 ± 0.13	1.05 ± 0.07	0.000 *
**ApEn**	0.85 ± 0.42	0.48 ± 0.29	1.08 ± 0.43	0.51 ± 0.19	0.000 *
**SampEn**	1.72 ± 0.23	1.48 ± 0.30	0.94 ± 0.39	1.29 ± 0.19	0.000 *
**FuzzyEn**	2.63 ± 0.29	2.31 ± 0.42	1.53 ± 0.43	2.08 ± 0.23	0.000 *
**PeEn**	2.52 ± 0.06	2.49 ± 0.06	2.54 ± 0.04	2.50 ± 0.04	0.000 *
**Complexity_1–4_**	6.08 ± 0.81	6.32 ± 1.04	3.99 ± 1.53	5.61 ± 0.72	0.000 *
**Complexity_1–20_**	27.47 ± 4.42	33.62 ± 4.88	23.95 ± 6.65	30.48 ± 3.63	0.000 *

**Table 4:** Variability of HRV in different time periods during free-running daily activities (data was presented as mean ± standard deviation). * *p* < 0.05.

	Morning (n = 50)	Noon (n = 49)	Evening (n = 51)	*p*
**Num-rr**	4886.50 ± 743.77	4962.10 ± 562.09	4869.39 ± 642.39	0.755
**RR-mean**	718.34 ± 86.67	725.07 ± 82.57	727.86 ± 96.37	0.859
**RR-median**	717.67 ± 90.38	726.35 ± 86.41	733.51 ± 104.11	0.699
**SDNN**	99.74 ± 41.72	86.33 ± 28.85	93.57 ± 42.13	0.220
**RMSSD**	44.68 ± 42.64	38.05 ± 12.50	49.77 ± 44.04	0.485
**SDSD**	44.68 ± 42.65	38.06 ± 12.51	49.77 ± 44.04	0.485
**SDANN**	66.63 ± 34.48	57.46 ± 33.46	55.38 ± 31.92	0.201
**NN50**	720.66 ± 668.94	731.57 ± 450.86	827.71 ± 568.43	0.585
**pNN50**	15.31 ± 13.47	15.41 ± 10.20	18.04 ± 13.76	0.469
**TP**	877,506.52 ± 358,835.21	855,094.12 ± 243,956.73	888,768.21 ± 229,642.28	0.834
**VLF**	9845.47 ± 8974.84	7926.83 ± 4530.85	9557.84 ± 6391.30	0.331
**LF**	4679.59 ± 4804.97	3581.90 ± 1917.09	4381.01 ± 4258.90	0.924
**HF**	1833.74 ± 2495.58	1547.95 ± 1247.57	1724.11 ± 1911.60	0.765
**LF-HF-ratio**	3.58 ± 2.13	2.92 ± 1.41	3.25 ± 2.02	0.222
**LF- percent**	33.74 ± 1.30	33.61 ± 0.91	33.54 ± 1.20	0.676
**HF- percent**	29.02 ± 1.87	29.52 ± 1.45	29.28 ± 1.71	0.342
**DFA-alpha1**	1.19 ± 0.20	1.18 ± 0.14	1.14 ± 0.22	0.358
**DFA-alpha2**	1.04 ± 0.10	1.03 ± 0.09	1.04 ± 0.11	0.759
**ApEn**	0.53 ± 0.29	0.52 ± 0.27	0.51 ± 0.27	0.950
**SampEn**	1.17 ± 0.30	1.41 ± 0.33	1.37 ± 0.34	0.001 *
**FuzzyEn**	1.97 ± 0.37	2.20 ± 0.38	2.16 ± 0.42	0.009 *
**PeEn**	2.49 ± 0.06	2.51 ± 0.05	2.52 ± 0.05	0.027 *
**Complexity_1–4_**	5.26 ± 1.13	6.04 ± 1.16	5.76 ± 1.23	0.005 *
**Complexity_1–20_**	30.03 ± 5.20	31.98 ± 4.74	30.43 ± 5.77	0.152

**Table 5:** Variability of HRV from sitting and lying posture (data was presented as mean ± standard deviation). * *p* < 0.05.

	Sitting (n = 51)	Lying (n = 50)	*p*
**Num-rr**	4602.16 ± 601.85	4945.32 ± 1394.07	0.114
**RR-mean**	774.62 ± 97.27	864.22 ± 112.48	0.000 *
**RR-median**	779.89 ± 99.18	877.40 ± 120.98	0.000 *
**SDNN**	90.29 ± 38.44	117.89 ± 38.75	0.001 *
**RMSSD**	55.88 ± 56.97	74.01 ± 48.06	0.087
**SDSD**	55.88 ± 56.97	74.02 ± 48.07	0.087
**SDANN**	44.10 ± 21.24	63.99 ± 29.48	0.000 *
**NN50**	962.53 ± 659.97	1562.64 ± 790.25	0.000 *
**pNN50**	22.09 ± 15.76	32.96 ± 16.34	0.001 *
**TP**	989,060.80 ± 343,791.86	2,395,637.74 ± 1,409,513.55	0.000 *
**VLF**	11,827.35 ± 10,470.17	24,402.23 ± 16,106.63	0.000 *
**LF**	5393.98 ± 5207.24	9570.96 ± 7491.56	0.002 *
**HF**	2393.98 ± 2402.09	5863.54 ± 5783.15	0.000 *
**LF-HF-ratio**	2.77 ± 1.78	2.08 ± 1.09	0.021 *
**LF- percent**	33.34 ± 1.12	32.91 ± 0.77	0.027 *
**HF- percent**	29.87 ± 1.63	30.62 ± 1.59	0.021 *
**DFA-alpha1**	1.12 ± 0.21	1.04 ± 0.19	0.054
**DFA-alpha2**	1.05 ± 0.10	1.07 ± 0.11	0.440
**ApEn**	0.48 ± 0.29	0.31 ± 0.23	0.002 *
**SampEn**	1.48 ± 0.30	1.52 ± 0.31	0.564
**FuzzyEn**	2.31 ± 0.42	2.51 ± 0.34	0.008 *
**PeEn**	2.49 ± 0.06	2.53 ± 0.05	0.000 *
**Complexity_1–4_**	6.32 ± 1.04	6.00 ± 1.11	0.135
**Complexity_1–20_**	33.62 ± 4.88	30.11 ± 5.33	0.001 *

**Table 6:** HRV metrics analyzed for different lying postures (data was presented as mean ± standard deviation). * *p* < 0.05.

	Left (n = 27)	Right (n = 27)	Back (n = 42)	*p*
**Num-rr**	2161.37 ± 514.58	2120.67 ± 593.06	2914.69 ± 993.90	0.000 *
**RR-mean**	869.05 ± 107.37	891.25 ± 111.12	849.15 ± 126.95	0.349
**RR-median**	879.28 ± 118.93	901.70 ± 124.88	861.81 ± 134.61	0.451
**SDNN**	99.89 ± 40.50	115.24 ± 52.27	111.45 ± 40.90	0.409
**RMSSD**	66.66 ± 43.46	78.58 ± 63.12	73.28 ± 57.02	0.732
**SDSD**	66.68 ± 43.48	78.60 ± 63.13	73.29 ± 57.05	0.732
**SDANN**	46.16 ± 36.49	54.61 ± 42.69	57.83 ± 29.40	0.410
**NN50**	676.44 ± 395.02	771.04 ± 422.57	871.76 ± 542.83	0.245
**pNN50**	32.38 ± 18.54	36.99 ± 17.69	31.21 ± 18.28	0.423
**TP**	900,278.49 ± 678,316.58	939,125.30 ± 616,280.79	1,005,243.53 ± 594,231.80	0.781
**VLF**	8448.99 ± 6180.05	14,168.82 ± 16,007.05	11,568.04 ± 8953.77	0.311
**LF**	3464.51 ± 2537.91	6113.47 ± 9382.50	4890.84 ± 4069.96	0.345
**HF**	2226.48 ± 1971.76	3967.30 ± 7362.43	3110.64 ± 3034.31	0.655
**LF-HF-ratio**	2.10 ± 1.13	2.12 ± 0.97	2.33 ± 1.51	0.712
**LF- percent**	32.87 ± 0.91	32.68 ± 0.97	32.99 ± 1.07	0.456
**HF- percent**	30.35 ± 2.09	30.01 ± 1.89	30.24 ± 2.15	0.833
**DFA-alpha1**	1.06 ± 0.20	1.03 ± 0.22	1.05 ± 0.21	0.833
**DFA-alpha2**	1.07 ± 0.11	1.09 ± 0.13	1.06 ± 0.13	0.651
**ApEn**	0.19 ± 0.19	0.13 ± 0.12	0.23 ± 0.18	0.065
**SampEn**	1.65 ± 0.25	1.60 ± 0.30	1.52 ± 0.32	0.192
**FuzzyEn**	2.55 ± 0.31	2.58 ± 0.36	2.48 ± 0.36	0.536
**PeEn**	2.53 ± 0.05	2.54 ± 0.05	2.52 ± 0.05	0.535
**Complexity_1–4_**	6.48 ± 0.86	6.20 ± 0.91	6.09 ± 1.06	0.249
**Complexity_1–10_**	16.20 ± 2.17	15.56 ± 2.34	15.34 ± 2.56	0.340

**Table 7:** Male and female HRV analysis and comparison results table (data was presented as mean ± standard deviation). * *p* < 0.05.

		Sleeping	Morning (FreeDA)	Sitting (Reading)	Lying (Reading)	Noon (FreeDA)	Brisk Walking	Evening (FreeDA)
**Num-rr**	Man	3276.93 ± 357.44	4707.07 ± 811.94	4412.07 ± 609.25	4795.88 ± 1300.25	4696.31 ± 383.53	3856.81 ± 771.09	4643.36 ± 522.01
	Woman	3725.67 ± 651.09	5097.13 ± 605.91	4816.00 ± 526.98	5107.21 ± 1500.02	5262.57 ± 586.77	4606.69 ± 777.37	5144.57 ± 677.70
	*p*	0.005 *	0.064	0.015 *	0.436	0.000 *	0.001 *	0.004 *
**RR-mean**	Man	1079.98 ± 106.03	746.97 ± 91.40	808.66 ± 102.30	898.11 ± 118.05	767.47 ± 71.03	620.78 ± 76.34	761.13 ± 99.87
	Woman	931.80 ± 120.62	684.74 ± 68.36	736.32 ± 76.46	827.51 ± 95.41	677.13 ± 67.93	537.97 ± 86.27	687.35 ± 75.79
	*p*	0.000 *	0.010 *	0.007 *	0.025 *	0.000 *	0.001 *	0.005 *
**RR-median**	Man	1095.25 ± 109.50	747.54 ± 99.37	816.69 ± 103.63	914.42 ± 127.92	767.85 ± 78.00	601.91 ± 81.19	769.80 ± 109.46
	Woman	934.61 ± 126.43	682.61 ± 64.38	738.50 ± 76.59	837.29 ± 100.82	679.43 ± 71.02	524.15 ± 90.52	689.33 ± 78.82
	*p*	0.000 *	0.010 *	0.004 *	0.023 *	0.000 *	0.002 *	0.005 *
**SDNN**	Man	104.23 ± 27.80	113.96 ± 42.44	103.72 ± 46.20	127.09 ± 40.57	92.61 ± 32.38	79.16 ± 41.34	107.45 ± 48.30
	Woman	80.70 ± 28.66	83.04 ± 34.76	75.18 ± 17.86	107.92 ± 34.80	79.23 ± 22.92	64.47 ± 36.51	76.67 ± 25.06
	*p*	0.004 *	0.008 *	0.007 *	0.080	0.106	0.177	0.006 *
**RMSSD**	Man	72.79 ± 29.00	53.82 ± 55.37	68.09 ± 75.21	78.96 ± 60.41	39.19 ± 10.43	26.85 ± 15.75	62.23 ± 55.37
	Woman	65.11 ± 29.81	33.94 ± 14.28	42.14 ± 16.97	68.65 ± 29.96	36.77 ± 14.64	23.72 ± 23.45	34.60 ± 14.37
	*p*	0.352	0.082	0.105	0.454	0.505	0.569	0.016 *
**SDSD**	Man	72.80 ± 29.00	53.83 ± 55.37	68.10 ± 75.22	78.97 ± 60.41	39.19 ± 10.44	26.86 ± 15.75	62.24 ± 55.38
	Woman	65.12 ± 29.82	33.95 ± 14.28	42.14 ± 16.97	68.66 ± 29.96	36.77 ± 14.64	23.72 ± 23.46	34.60 ± 14.37
	*p*	0.352	0.082	0.105	0.454	0.505	0.569	0.016 *
**SDANN**	Man	53.00 ± 25.07	74.02 ± 31.98	47.08 ± 25.33	64.77 ± 29.18	60.80 ± 38.18	63.09 ± 42.38	61.08 ± 35.39
	Woman	37.47 ± 17.52	57.96 ± 35.96	40.75 ± 15.27	63.14 ± 30.41	53.68 ± 27.53	50.17 ± 37.30	48.43 ± 26.20
	*p*	0.012 *	0.101	0.292	0.847	0.463	0.245	0.161
**NN50**	Man	1380.00 ± 582.14	850.59 ± 820.56	1110.33 ± 738.74	1497.73 ± 686.41	750.92 ± 398.49	236.56 ± 246.09	979.64 ± 591.03
	Woman	1299.29 ± 627.46	568.13 ± 394.84	796.25 ± 524.83	1632.96 ± 899.11	709.70 ± 511.98	205.35 ± 279.26	642.74 ± 490.41
	*p*	0.633	0.138	0.090	0.551	0.753	0.668	0.034 *
**pNN50**	Man	43.38 ± 19.49	18.35 ± 15.98	26.33 ± 17.41	32.69 ± 16.12	16.45 ± 9.45	6.70 ± 7.58	21.99 ± 14.99
	Woman	36.26 ± 19.53	11.75 ± 8.80	17.33 ± 12.35	33.25 ± 16.91	14.23 ± 11.08	4.94 ± 7.09	13.23 ± 10.51
	*p*	0.195	0.072	0.041 *	0.905	0.453	0.387	0.022 *
**TP**	Man	1,860,802.31 ± 371,337.19	984,906.23 ± 437,538.33	1,102,124.05 ± 406,070.18	2,523,628.42 ± 1,407,752.56	963,705.06 ± 232,973.27	713,373.60 ± 327,305.49	975,248.48 ± 225,058.10
	Woman	1,438,320.34 ± 431,990.30	751,428.60 ± 172,534.79	861,864.64 ± 196,599.66	2,256,981.17 ± 1,428,257.91	732,316.53 ± 196,143.10	618,700.08 ± 287,625.79	783,487.88 ± 191,270.82
	*p*	0.000 *	0.015 *	0.011 *	0.510	0.001 *	0.269	0.002 *
**VLF**	Man	13,803.55 ± 7755.53	12,521.56 ± 10,803.76	16,719.86 ± 12,214.49	30,402.21 ± 18,226.87	9881.73 ± 4844.70	4968.93 ± 6312.48	11,674.82 ± 7322.63
	Woman	9297.67 ± 11,463.08	6703.98 ± 4727.77	6323.26 ± 3154.14	17,902.24 ± 10,334.18	5716.94 ± 2914.77	4824.98 ± 10,185.00	6980.65 ± 3792.46
	*p*	0.099	0.016 *	0.000 *	0.004 *	0.001 *	0.951	0.008 *
**LF**	Man	5109.36 ± 2928.40	6152.54 ± 5901.79	7544.14 ± 6149.00	11,400.63 ± 8214.23	4333.31 ± 1970.92	2338.24 ± 3146.62	5766.09 ± 5054.16
	Woman	4465.95 ± 5056.84	2950.46 ± 2114.31	2975.05 ± 2161.38	7588.81 ± 6192.12	2732.48 ± 1477.44	2383.70 ± 7060.87	2694.82 ± 2111.65
	*p*	0.570	0.013 *	0.001 *	0.072	0.003 *	0.976	0.009 *
**HF**	Man	4507.65 ± 3374.09	2250.66 ± 3168.09	3043.96 ± 2986.95	5786.14 ± 6004.52	1580.03 ± 1118.57	742.87 ± 986.40	1971.24 ± 2287.27
	Woman	4675.38 ± 4007.69	1344.31 ± 1243.33	1662.75 ± 1184.92	5947.39 ± 5661.13	1511.68 ± 1404.08	514.19 ± 825.16	1423.26 ± 1308.47
	*p*	0.870	0.204	0.033 *	0.923	0.851	0.365	0.313
**LF-HF-ratio**	Man	1.54 ± 0.94	3.89 ± 2.01	3.15 ± 1.79	2.49 ± 1.20	3.37 ± 1.50	4.40 ± 2.19	3.71 ± 2.15
	Woman	1.08 ± 0.67	3.22 ± 2.25	2.34 ± 1.69	1.64 ± 0.75	2.41 ± 1.13	4.26 ± 2.89	2.70 ± 1.75
	*p*	0.049 *	0.276	0.104	0.005 *	0.016 *	0.840	0.076
**LF- percent**	Man	32.30 ± 1.26	33.92 ± 1.04	33.56 ± 1.03	33.07 ± 0.70	33.81 ± 1.04	34.21 ± 1.21	33.92 ± 1.14
	Woman	31.93 ± 1.37	33.53 ± 1.55	33.09 ± 1.19	32.73 ± 0.81	33.39 ± 0.70	33.62 ± 1.44	33.08 ± 1.13
	*p*	0.317	0.322	0.136	0.118	0.102	0.107	0.122
**HF- percent**	Man	31.52 ± 2.12	28.78 ± 1.67	29.55 ± 1.41	30.09 ± 1.44	29.16 ± 1.16	27.41 ± 2.68	29.13 ± 1.40
	Woman	32.54 ± 2.09	29.31 ± 2.08	30.23 ± 1.82	31.20 ± 1.57	29.93 ± 1.65	26.84 ± 2.90	29.45 ± 2.06
	*p*	0.086	0.322	0.136	0.012 *	0.062	0.457	0.535
**DFA-alpha1**	Man	0.97 ± 0.22	1.22 ± 0.21	1.16 ± 0.25	1.11 ± 0.19	1.22 ± 0.13	1.24 ± 0.20	1.14 ± 0.25
	Woman	0.88 ± 0.17	1.16 ± 0.18	1.08 ± 0.16	0.97 ± 0.17	1.12 ± 0.14	1.24 ± 0.17	1.14 ± 0.18
	*p*	0.086	0.318	0.181	0.010 *	0.013 *	0.876	0.955
**DFA-alpha2**	Man	1.14 ± 0.10	1.04 ± 0.11	1.04 ± 0.11	1.09 ± 0.10	1.03 ± 0.08	1.02 ± 0.13	1.02 ± 0.11
	Woman	1.08 ± 0.08	1.04 ± 0.10	1.06 ± 0.10	1.04 ± 0.11	1.03 ± 0.09	1.06 ± 0.13	1.07 ± 0.10
	*p*	0.019 *	0.960	0.399	0.151	0.874	0.272	0.064
**ApEn**	Man	0.77 ± 0.34	0.44 ± 0.25	0.42 ± 0.31	0.27 ± 0.18	0.43 ± 0.19	0.95 ± 0.41	0.39 ± 0.17
	Woman	0.94 ± 0.49	0.63 ± 0.32	0.54 ± 0.24	0.35 ± 0.28	0.61 ± 0.32	1.22 ± 0.43	0.65 ± 0.32
	*p*	0.162	0.019 *	0.135	0.230	0.017 *	0.024 *	0.002 *
**SampEn**	Man	1.72 ± 0.25	1.12 ± 0.29	1.45 ± 0.36	1.41 ± 0.27	1.41 ± 0.31	1.00 ± 0.36	1.31 ± 0.38
	Woman	1.72 ± 0.19	1.23 ± 0.30	1.52 ± 0.225	1.63 ± 0.32	1.41 ± 0.35	0.87 ± 0.41	1.43 ± 0.27
	*p*	0.938	0.204	0.374	0.010 *	0.942	0.222	0.202
**FuzzyEn**	Man	2.69 ± 0.28	1.96 ± 0.42	2.30 ± 0.52	2.45 ± 0.31	2.24 ± 0.32	1.65 ± 0.37	2.12 ± 0.47
	Woman	2.55 ± 0.30	1.98 ± 0.31	2.31 ± 0.29	2.58 ± 0.37	2.16 ± 0.44	1.42 ± 0.46	2.20 ± 0.35
	*p*	0.096	0.863	0.888	0.176	0.456	0.049 *	0.495
**PeEn**	Man	2.53 ± 0.04	2.49 ± 0.06	2.49 ± 0.07	2.53 ± 0.05	2.50 ± 0.05	2.53 ± 0.04	2.52 ± 0.05
	Woman	2.50 ± 0.07	2.49 ± 0.05	2.49 ± 0.05	2.53 ± 0.04	2.51 ± 0.04	2.55 ± 0.03	2.51 ± 0.04
	*p*	0.046 *	0.929	0.772	0.624	0.192	0.013 *	0.477
**Complexity_1–4_**	Man	5.97 ± 0.91	5.10 ± 1.09	6.25 ± 1.24	5.76 ± 1.04	6.18 ± 1.12	4.35 ± 1.37	5.60 ± 1.36
	Woman	6.21 ± 0.67	5.45 ± 1.18	6.40 ± 0.78	6.26 ± 1.15	5.88 ± 1.22	3.61 ± 1.61	5.95 ± 1.05
	*p*	0.311	0.281	0.610	0.114	0.374	0.080	0.321
**Complexity_1–10_**	Man	14.15 ± 2.34	14.10 ± 2.71	16.35 ± 2.89	14.63 ± 2.69	16.18 ± 2.56	12.13 ± 3.30	14.69 ± 3.33
	Woman	14.78 ± 1.66	14.82 ± 2.63	16.59 ± 1.96	15.35 ± 2.61	15.33 ± 2.56	10.22 ± 3.75	15.39 ± 2.36
	*p*	0.276	0.349	0.734	0.342	0.251	0.054	0.398
**Complexity_1–20_**	Man	26.76 ± 4.90	29.51 ± 5.44			32.56 ± 4.73	25.56 ± 6.17	29.70 ± 6.64
	Woman	28.31 ± 3.72	30.63 ± 4.96			31.34 ± 4.76	22.28 ± 6.83	31.32 ± 4.46
	*p*	0.211	0.454			0.373	0.072	0.322
